# General Health in the Elderly and Younger Adults of Rural areas in Fars Province, Iran

**Published:** 2015-01

**Authors:** Najaf Zare, Farkhondeh Sharif, Tania Dehesh, Fariba Moradi

**Affiliations:** 1 Department of Biostatistics, Infertility Research Center, Shiraz University of Medical Sciences, Shiraz, Iran;; 2 Community Based Psychiatric Care Research Center, Department of Mental Health and Psychiatric Nursing, School of Nursing and Midwifery, Shiraz University of Medical Sciences, Shiraz, Iran;; 3 Department of Biostatistics, Shiraz University of Medical Sciences, Shiraz, Iran;; 4 Family Health Unit, Shiraz University of Medical Sciences, Shiraz, Iran

**Keywords:** Elderly, General Health, Iran, Mental Disorder, Rural Area

## Abstract

**Background:** There are critical gaps in assessment and research on health among the elderly living in rural communities. The **s**tate of aging and health in rural areas provides a snapshot of our older adults’s need to necessary public health measures .The aim of this study was to determine the self-rated general health of adults residing in rural areas and compare the general health of the elderly with younger adults.

**Methods:** In this population based study using multistage random sampling, 2259 adults aged (≥15 years old) were selected from rural areas of Shiraz, southern Iran. The participants were divided into three age groups: young adults (15-39 years old n=1574), middle aged adults (40-59 years old, n=530), and the elderly (≥60 years of age, n-155).  Data were gathered using a translated version of the general health questionnaire (GHQ-28) and analyzed using Chi-square, one-way ANOVA, Kruskal-Wallis tests and ANCOVA analysis.  SPSS software, version 16, was used for analysis.

Results:  34.8%, 31.6%, 52.3% and 7.7% of the elderly had a probable mental disorder  in the somatization, anxiety, social dysfunction and depression categories, respectively. Moreover, 9.7, 7.1, 3.9 and 4.5% of the elderly had a  severe mental disorder  in the four mentioned subscales, respectively. Compared with younger adults, the elderly showed a significantly higher disorder in all subscales except for anxiety.

Conclusion: Our findings showed that chronic disease had a great effect on general health. Screening programs and prevention of chronic disease by the newly established family physician in rural districts can improve the overall community health.

## Introduction

Today, dealing with problems and diseases of the elderly people is crucial. Late adulthood is a period of life which begins at the age of 60-65. The World Health Organization (WHO) defines individuals aged ≥60 as the elderly.^1^ The population of the elderly people is increasing because of reduced mortality rates, increased life expectancy and declining birth rate. This phenomenon results in elderly disease and disorders.

The cost of physical illness in the elderly is increasing. These patients have to pay to the specialists instead of a general practitioner. In general, mental disorders such as anxiety and depression are risk factors for many diseases such as diabetes, coronary artery diseases and CVA that creates financial constraints for the elderly.^2^^,^^3^ Alternatively, individuals with one or more chronic illnesses often experience a number of psychological disorders, ranging from depressive symptoms to major psychiatric disorders, because of their direct hormonal or neural endocrine effects or as a side effect of drug therapy.^4^ Depression is one of the most ageing diseases that has a huge health care cost burden and researchers  believe that it will have the highest burden of cost in the old population  in 2020.^2^

Therefore, screening and treatment of mental disorders in the early stages of mental disorders reduce these costs and will lead to a healthier community. Community-based screening for depression and other mental disorders is a valuable tool for identification of ill individuals.

The prevalence of health-related problems differs between the elderly and younger adults because of the different characteristics of the two age groups. In a population-based study in Australia, 17.7% of the participants had one or more common mental disorders.^5^ In a study performed in Iran, education level, sex and marital status were associated with the risk of depression.^6^

Many tools have been proposed for screening general health and mental disorders. Screening questionnaires are generally easy-to-perform tools used to identify individuals who are likely to suffer from some disorders.^6^^,^^7^ The instruments used during the screening stage should have adequate sensitivity and specificity. The General Health Questionnaire (GHQ), designed by Goldberg,^8^ is a self-administered screening tool designed to detect non-psychotic psychiatric disorders in primary care settings and in the community. The short version of this questionnaire (GHQ-28) has been translated, validated and used in different populations.^9^^,^^10^ The Persian version of the GHQ-28 has been validated in the elderly population.^10^

We aimed to compare the general health (specifically, mental disorder) in the elderly and younger adults and estimate the confounding effects of some variables on GHQ scores in this particular population. 

## Materials and Methods

In this population-based study, 2259 adults aged ≥15 years consisting of 155 elderly (≥60 years of age) and 2104 younger adults were selected from rural areas of Shiraz (southern Iran) using multistage random sampling. They were selected from a list of village Health Houses in 2008. On the first stage, 18 Health Houses were selected; then in the second stage, the subjects were selected randomly within each Health House. Data were collected by health workers (Behvarz) who had full knowledge of the study population. The participants were divided into three age groups: young adults (15-39 years old, n=1574, middle age adults (40-59 years old, n=530), and the elderly (≥60 years old age, n=155). Demographic characteristics of these three age groups were recorded. The education level in rural areas is not drastically different. So, we divided the people into two groups of illiterate (cannot read and write) and literate (some knowledge of  reading and writing). Chronic disease is a long-lasting condition that can be controlled, but not cured, such as hypertension, diabetes mellitus, skeletal chronic pain, etc. A person’s marital status indicates whether the person is now married or not (like separated,  single and divorced).

Data gathering was done using a Persian version of the GHQ-28 questionnaire.  It has 28 items that contain four subscales of somatic symptoms, anxiety/insomnia, social dysfunction, and depression. Each of these four subscales contains 7 items scored on a Likert scale. The GHQ-28 has a 4-item response with ‘Not at all’, ‘No more than usual’, ‘Rather more than usual’, and ‘Much more than usual’. Several scoring methods are available; we used the Likert scale to show the symptoms’ severity with scores between 0–3 (0–1–2–3, subscale range 0 to 21). A greater score indicates lower health.  Since the optimal threshold concept is more useful for estimating the prevalence in large population than screening for individual cases, we classified the participants using the cutoff point of 7 for probable mental disorder and 14 for  severe mental disorder in each domain and 23 for the total GHQ score, as suggested for the Iranian version by Shahrokhi.^11^ Data were obtained from a university approved project and all ethical issues were considered. The subjects  who did not agree to participate in this research were excluded.

*Statistical Methods*

Analysis of covariance (ANCOVA) provides a more complete picture of the conditional distribution of Y given X=x when the covariates are a mixture of continues and categorical variables. In fact ANCOVA is an analysis of variances when some continuous covariate added to the analysis of variance (ANOVA) for controlling their effects, besides other important covariates. In ANCOVA model, the continuous covariates are not very crucial for the researchers, but their effects on the important covariates (usually categorical variables) must be controlled. Hence, we used the ANCOVA approach to explore a more complete picture of covariate effects.^12^^,^^13^

The research interest may be an outcome variable that takes the value within a defined range. For instance, GHQ and subscale scores are bounded in the interval 0-84 and 0-21, respectively. More examples can be found in many medical disciplines. In large sampling, this restriction is not crucial. Consider a continuous outcome variable y and a set of covariates x={x_1_, x_2_, … x_k_} and consider that some of them are continuous and the others are categorical variables. The ANCOVA model is: y=β_0_+β_1_x_1_+β_2_x_2_+…+β_k_x_k_. ANCOVA is designed to control for covariates. In fact like ANOVA, categorical covariates have a more important role in the result and continuous covariates exist for reducing the bias of the results. ANCOVA is an excellent method for comparing changes between groups; without ANCOVA analysis, the results will yield biased conclusions.^13^ In each ANCOVA process, we controlled one of the demographic variables and showed the influence of other covariate on each subscale score.

Analysis was done using Chi-square, t-test, ANOVA, Kruskal-Wallis test, and ANCOVA model, using SPSS software, version 16. The Kruskal-Wallis is a nonparametric test which is used for comparing the means of more than two groups, like ANOVA in parametric tests. The P values are compared with .05. If their P value is less than. 05, then the test is rejected. ANCOVA is a kind of regression model, so the result of coefficients is used for comparison. 

## Results

Of the 2313 participants, 6.9% were elderly, 23.4% middle aged, and 69.6% young adults.  56.8% were women and 43.2% men. About 2-3% missing values were present in some variables. In each stage, analysis was done with all available data. The distribution of demographic characteristics are different in three age groups and shown in Table 1.

**Table 1:** Demographic characteristics of the three age groups in rural areas of Shiraz

**Variables**		**Elderly** **n (%)**	**Middle-aged adults** **n (%)**	**Young adults** **n (%)**	**P value**
Sex	M	89 (57.8)	266 (50.6)	614 (39.0)	<0.001
	F	65 (42.2)	260 (49.4)	959 (61.0)	
Education	Illiterate	47 (66.2)	73 (18.0)	33 (2.2)	<0.001
	Literate	24 (33.8)	332 (82.0)	1485 (97.8)	
Marital status	Married	117 (76.0)	490 (92.5)	1507 (96.0)	<0.001
	Else	37 (24.0)	40 (7.5)	63 (4.0)	
Chronic disease	Yes	76 (49.0)	411 (77.5)	1401 (89.2)	<0.001
	No	79 (51.0)	119 (22.5)	170 (10.8)	

We found a significant difference between the total mean scores and the mean scores of the different subscales of the GHQ-28 in the three age groups. However, there was no significant difference between the young and middle aged groups in all subscales. The elderly had lower heath quality (Table 2).

**Table 2:** Mean±SD scores of the GHQ-28 subscales in the three age groups

**GHQ-28 subscales**	**Elderly** **(n=155)**	**Middle-aged adults** **(n=530)**	**Young adults** **(n=1574)**	**P value**
Somatic symptoms	6.8±4.5	5.78±3.8	5.4±3.7	<0.001
Anxiety	5.8±4.5	5.2±4.4	4.9±4.2	0.030
Social dysfunction	7.6±2.8	6.9±2.0	6.7±2.2	<0.001
Depression	2.7±4.1	2.0±3.0	2.1±3.2	0.050
Total score	22.9±13.5	19.9±10.7	19.2±10.9	<0.001

34.8, 31.6, 52.3 and 7.7% of the elderly had probable mental disorder in somatic symptoms, anxiety, social dysfunction and depression subscales, respectively. Moreover, 9.7, 7.1, 3.9 and 4.5% of the elderly had  severe mental disorder in the four mentioned subscales, respectively. Compared with younger adults, the elderly showed significantly higher disorders in all subscales except anxiety (Table 3).

**Table 3:** The frequency (%) of normal, probable, and  severe mental disorder in the three age groups with respect to different subscales of the GHQ-28

**Subscales**	**Elderly** **(n=155)**	**Middle-aged adults** **(n=530)**	**Young adults** **(n=1574)**	**P value**
Somatic symptoms				
Normal	86 (55.5)	333 (62.8)	1078 (68.5)	<0.001
Probable mental disorder l	54 (34.8)	177 (33.4)	438 (27.8)	
Severe mental disorder l	15 (9.7)	20 (3.8)	58 (3.7)	
Anxiety				
Normal	95 (61.3)	346 (65.3)	1067 (67.8)	0.195
Probable mental disorder l	49 (31.6)	164 (30.9)	445 (28.3)	
Severe mental disorder l	11 (7.1)	20 (3.8)	62 (3.9)	
Social dysfunction				
Normal	68 (43.9)	234 (44.2)	757 (48.3)	0.026
Probable mental disorder	81 (52.3)	290 (54.7)	784 (50.1)	
Severe mental disorder	6 (3.9)	6 (1.1)	25 (1.6)	
Depression				
Normal	136 (87.7)	479 (90.4)	1403 (89.1)	0.006
Probable mental disorder	12 (7.7)	46 (8.7)	148 (9.4)	
Severe mental disorder	7 (4.5)	5 (0.9)	23 (1.5)	
Total				
Normal	96 (61.9)	353 (66.6)	1068 (67.9)	0.289
Disorder	59 (38.1)	177 (33.4)	506 (32.1)	

Sex distribution (male=0, female=1), chronic disease (healthy=0, ill=1), marital status (married=1, else=0), and education (number of years) were different in the three age groups. To see the effect of senility on total GHQ-28 and subscale scores, we controlled these covariates in Ancova model. 

After controlling these covariates, the result of Ancova model showed that marital status and education do not have a significant effect on somatic symptoms, anxiety  subscales and total CHQ health, but the other covariates had a significant effect.

Sex and marital status and education did not show a significant effect on social and depression subscales, but the other covariates had significant effects, like other subscales. The only difference between the subscales in covariates effect was sex. The other covariates showed similar effects on total and subscales scores.

**Figure 1:**
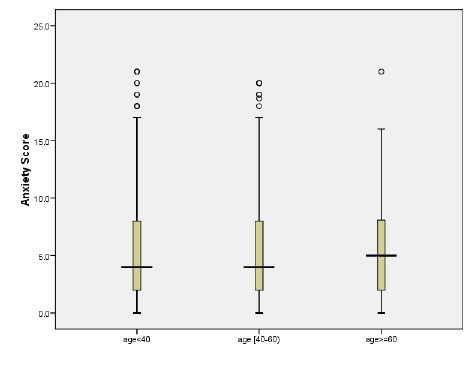
Comparison of anxiety score distribution among the three age groups

Men and women of all age groups did not differ in the mean scores of social dysfunction and depression subscales. However, women had significantly higher scores in the somatic symptoms subscales in three age groups, indicating lower general health. Younger adults had significantly higher mean anxiety score compared with the other age groups (Figure 1).

Education level had a significant effect on the mean score of somatic symptoms in all age groups. Elderly people with chronic disease showed a significantly lower health status in general health and other subscales except depression.  

## Discussion

We found that the elderly had a higher total as well as subscale scores of general health. Moreover, based on the cut-off points of 23 and 7 for general health and subscales, the prevalence of probable and  severe mental disorder of general health, anxiety and depression were 31.9, 32.2, and 10.5%, respectively. Also, the history of chronic disease and sex had a substantial effect on the general health scores that reduced the effect of senility in multiple quantile logistic regression.  

The reported score of the GHQ in an Iranian population by Momeni  and colleagues showed that total and social dysfunction scores of the GHQ were nearly the same, but there were some discrepancies with other subscales.^3^ The history of chronic health problems in the elderly was correlated with symptoms of anxiety and depression in other studies.^6^^,^^14^ In a review, the prevalence of major depression ranged from 1-16% among the elderly living in private houses or in institutions,^15^ which is somehow similar to our results. Also, the prevalence of depression in an elderly population in Germany was reported to be 3.5%.^7^ Another study showed differences between men and women in the prevalence of mental disorder.^3^

Epidemiological studies have shown that depression is common among the elderly population, and the prevalence rate was very high in a systematic review of community–based studies (0.4-35%).^16^ In the study of Moradi et. al.,  the prevalence of depression in the elderly population of southern Iran was reported about 20% for severe depression;^17^ these  results are in the same with those of this study.  In this study consistent with others, the prevalence of depression symptoms increased with age.^18^^-^^23^ This may occur because of their reduced physical activity and the greater weakness of older people against stress and physical diseases. Woman had a higher prevalence rate of depression symptoms compared to men and this finding is similar to other reports from other countries.^19^^-^^21^^,^^23^ In fact, this report shows that this higher rate of depression in woman might be caused by some biological factors or their social pressure that they experience in the entire world.^21^^-^^23^ However, poor education and unemployment are associated with depression symptoms in women population.

One limitation of our study is its relatively small sample size of the elderly population, which creates difficulty in computing the prevalence of disorders. Larger studies on all rural areas  of the province are required to allow the impact of socio-demographic, chronic diseases and spatial variables on the health of the elderly population in rural areas. A proportion of subjects scoring above the corresponding cutoff score were false positives. Elevation of the cutoff score or variable cutoff for different subscales may reduce the misclassification rate of the GHQ-28. Methodological differences between the studies hinder consistent conclusions about geographical and cross-cultural variations in prevalence and predictors of mental disorders in the elderly population. Improved comparability will provide a basis for consistent conclusions. 

## Conclusion

Aging has a great effect on general health and puts people at increasing risk of some mental disorder. Screening programs for prevention of chronic disease and mental disorder  by the newly established family physician plan in the rural areas can improve the overall health in rural communities. 
